# Extracellular Cardiolipin Modulates Select Immune Functions of Astrocytes in Toll-Like Receptor (TLR) 4-Dependent Manner

**DOI:** 10.1155/2022/9946439

**Published:** 2022-03-25

**Authors:** Taryn E. Murray, Tyler J. Wenzel, Svetlana Simtchouk, Bridget K. Greuel, Julien Gibon, Andis Klegeris

**Affiliations:** Department of Biology, University of British Columbia Okanagan Campus, Kelowna, British Columbia, Canada V1V 1V7

## Abstract

Alzheimer's disease (AD) is characterized by chronic neuroinflammation, which is partially mediated by dysregulated functions of glial cells. Cardiolipin (CL) is a phospholipid normally confined to the inner mitochondrial membrane; however, it has been detected in human sera, indicating that it can exist in the extracellular space where it may interact with nearby cells. Although CL has been shown to modulate several functions of microglia in a toll-like receptor (TLR) 4-dependent manner, the effects of extracellular CL on astrocytes are unknown. In addition to their homeostatic functions, astrocytes participate in neuroimmune responses of the brain and express TLR 4. Therefore, we hypothesized that extracellular CL (1) modulates the secretion of cytokines and cytotoxins by astrocytes, as well as their phagocytic activity, and (2) acts by interacting with astrocyte TLR 4. We demonstrate that CL inhibits the lipopolysaccharide- (LPS-) induced secretion of cytotoxins and expression of glial fibrillary acidic protein (GFAP) by human U118 MG astrocytic cells. CL alone upregulates the phagocytic activity of human astrocytic cells and primary murine astrocytes. CL in combination with LPS upregulates secretion of interleukin (IL)-1*β* by astrocytic cells. Furthermore, CL alone increases the secretion of monocyte chemoattractant protein (MCP)-1 by astrocytic cells, which is blocked by the TLR 4-specific antagonist TAK-242. We demonstrate that CL upregulates MCP-1 secretion in the absence of its natural carrier protein, *β*2-glycoprotein 1, indicating that CL may be bioactive in the brain where this protein is not present. Lastly, we show that CL downregulates the expression of astrocytic TLR 4, implying that CL engages this receptor, as its activation has been shown to lead to its degradation. Overall, our study extends the list of cell type functions of which CL modulates and provides evidence that CL, or liposomes containing this phospholipid can be used to modulate specific neuroimmune functions of astrocytes.

## 1. Introduction

Alzheimer's disease (AD) is an incurable neurodegenerative disorder that clinically manifests as progressive cognitive decline and memory impairment. This disease is characterized by a state of chronic neuroinflammation, which is driven in part by dysfunctional nonneuronal glial cells [1]. Astrocytes are the most abundant type of glial cells in the central nervous system (CNS). They support brain homeostasis and are essential for neuron survival [2, 3]. In addition, astrocytes participate in the regulation of the immune response in the CNS, a role that has traditionally been attributed solely to microglia [4]. In AD brains, astrocyte functions become dysregulated due to the prolonged presence of aggregates of amyloid-*β* peptides and elevated concentrations of inflammatory mediators [5]. Such pathological conditions shift homeostatic astrocytes into a reactive state, characterized by the overexpression of glial fibrillary acidic protein (GFAP) and enhanced secretion of cytokines and cytotoxins [1, 3, 6–8]. Furthermore, the phagocytic capacity of reactive astrocytes has been reported to be impaired in AD [9]. Dysregulated astrocytic functions lead to damage of surrounding cells and tissues, contributing to the extensive death of neurons in AD brains. Therefore, the identification of molecules that can reduce this reactive state of astrocytes could facilitate the development of novel therapeutics effectively diminishing neuroinflammation in AD.

Cardiolipin (CL) is a mitochondrial phospholipid predominantly located in the inner mitochondrial membrane of mammalian cells. CL supports mitochondrial electron transport chain functionality and participates in the degradation of dysfunctional mitochondria and apoptotic processes [10, 11]. CL can relocate from the inner to the outer mitochondrial membrane where it acts as an elimination signal for dysfunctional mitochondria contributing to mitophagy and apoptosis [11–13]. In addition, CL has been detected in blood where it can be bound to the carrier protein *β*2-glycoprotein 1 [14], indicating that it exists outside cells. Although the origin of extracellular CL is still unknown, it has been proposed that CL can be released into the extracellular space from damaged or dying cells, where it can interact with receptors on nearby cells, including astrocytes [11, 15–17].

Toll-like receptor (TLR) 4 has been implicated as a molecular target for CL [16, 17]. TLR 4 belongs to the family of pattern recognition receptors that bind damage-associated molecular patterns (DAMPs) or pathogen-associated molecular patterns (PAMPs), playing an essential role in the innate immune response [18, 19]. TLR 4-mediated inflammatory mechanisms are involved in neurodegenerative diseases, including AD [18]. For example, the activation of TLR 4 triggers specific glial cell signaling pathways that upregulate the expression of genes encoding a broad range of inflammatory mediators, including the cytokine interleukin (IL)-1*β*, IL-6, tumor necrosis factor- (TNF-) *α*, interferon- (IFN-) *β*, monocyte chemoattractant protein- (MCP-) 1, and IFN-*γ*-induced protein- (IP-) 10 [20–22].

Previous studies have demonstrated the ability of CL to modulate TLR 4-dependent signaling in several different cell types. For example, CL-enriched liposomes promote phagocytosis and dampen cytokine secretion by primary human monocyte-derived macrophages and RAW264.7 murine macrophage-like cells [16]. Pointer et al. [15] demonstrate that CL decreases cytotoxicity of human THP-1 microglia-like cells towards human SH-SY5Y neuronal cells. Furthermore, this study reveals that CL decreases the release of TNF-*α* from IFN-*γ*- and lipopolysaccharide- (LPS-) stimulated THP-1 cells. A more recent study shows that CL induces the secretion of MCP-1 by murine BV-2 and human THP-1 microglia-like cells in a TLR 4-dependent manner [17]. In addition, CL upregulates the phagocytic activity of primary murine microglia and THP-1 microglia-like cells in a TLR 4-dependent manner. Since CL has been shown to interact with TLR 4 on other cell types and astrocytes express TLR 4 on their cell surface [16, 17, 23, 24], extracellularly released CL may interact with astrocyte TLR 4. Since no studies to date have demonstrated regulation of the immune functions of astrocytes by extracellular CL, we investigated the effects of this phospholipid on several functional parameters of astrocytes, including their GFAP expression, phagocytic activity, and secretion of cytokines and cytotoxins. In addition, we tested a hypothesis that the modulatory effects of extracellular CL on astrocytes are mediated by TLR 4.

## 2. Materials and Methods

### 2.1. Reagents

Bisbenzimide (Hoechst 33258), CL from bovine heart, cytochalasin B, cOmplete™ mini protease inhibitor cocktail, 1,4-dithiothreitol (DTT), 3-(4,5-dimethyl-2-thiazoyl)-2,5-diphenyl-2H-tetrazolium bromide (MTT), N-(1-naphthyl) ethylenediamine dihydrochloride, N,N-dimethylformamide, LPS from *Escherichia coli* 055: B5, poly-d-lysine, sigma 104 phosphatase substrate, TLR 4 antagonist TAK-242 (#614316), fluoroshield with 4′,6-diamidino-2-phenylindole (DAPI), and sodium deoxycholate were obtained from Sigma-Aldrich (Oakville, ON, Canada). Human recombinant IFN-*γ* and IL-1*β*, as well as enzyme-linked immunosorbent assay (ELISA) development kits for human IL-1*β*, MCP-1, and IP-10, were purchased from PeproTech (Embrun, ON, Canada). Anti-rabbit IgG horseradish peroxidase- (HRP-) linked antibody (#7074S) and a biotinylated protein ladder detection pack (#7727) were purchased from New England Biolabs (Ipswich, MA, USA). OptiLadder was purchased from Applied Biological Materials (Richmond, BC, Canada). Fluorescein isothiocyanate (FITC) externally labeled one *μ*m fluorescent polystyrene latex beads were purchased from Bangs Laboratories (Fishers, IN, USA). Texas red fluorophore-conjugated MemBrite Fix membrane stain, CellBrite™ Fix 555, and CellBrite™ Fix 640 were purchased from Biotium (Fremont, CA, USA). Anti-*β*-actin antibody I-19 (#SC-1616-R) was purchased from SantaCruz Biotechnology (Dallas, TX, USA). Rabbit anti-GFAP antibody (#Z0334) was purchased from Agilent (Santa Clara, CA, USA). Goat anti-rabbit IgG CY5- or CY3-labeled antibodies (#111-165-003) were purchased from Jackson ImmunoResearch (West Grove, PA, USA). Anti-mouse postsynaptic density protein (PSD)-95 antibody (#MA1-045), mouse anti-TLR 4 antibodies (#5015348; clone HTA125), and all other reagents were purchased from Fisher Scientific (Ottawa, ON, Canada).

### 2.2. Cell Culture Models

Human U118 MG cells were purchased from the American Type Culture Collection (ATCC, Manassas, VA, USA). They possess astroglioma morphology and thus were used as astrocyte models. Human neuroblastoma SH-SY5Y cells were donated by Dr. R. Ross (Department of Biological Sciences, Fordham University, Bronx, NY, USA). Cells were cultured in Dulbecco's Modified Eagle Medium Nutrient Mixture F-12 Ham (DMEM-F12) supplemented with 10% calf bovine serum (CBS), penicillin (100 U/ml), streptomycin (100 *μ*g/ml), and amphotericin B (500 ng/ml) in T-75 flasks incubated at 37°C in humidified 5% CO_2_ and 95% air atmosphere.

Animal experiment was approved by the University of British Columbia's Animal Care Committee and was performed in accordance with the Canadian Council on Animal Care standards. C57BL/6NCrl mice were received from Charles River Laboratory (Montreal, Quebec, Canada), maintained in the animal facility, and housed in sterilized, filter-topped cages. Efforts were made to reduce animal handling and use. Murine mixed cortical cell cultures were obtained from postnatal day one to four pups as previously described [25]. Briefly, cortices without meninges were collected in cold Hank's balance salt solution (HBSS) and trypsinized (0.25% trypsin in HBSS) at 37°C for 20 min. DNase I was added to the suspension for 15 min. The suspension was centrifuged for five min at 400 g, and the supernatant was discarded. The pellet was resuspended vigorously to obtain a single-cell suspension in DMEM containing 10% fetal bovine serum (FBS) and penicillin/streptomycin. Cells were transferred to a poly-D-lysine coated T-75 flask, incubated at 37°C, 5% CO_2,_ and media changed every two to three days. At eight days in vitro, the T-75 flask was shaken at 180 rpm for 60 min to detach the microglia. The media containing microglia were discarded, and fresh media added. The T-75 flask was then shaken at 240 rpm for six h to separate oligodendrocyte precursor cells. The media were removed, the T-75 flask washed twice with warm PBS, and new media were added to the astrocyte layer. The culture of astrocytes was maintained at 37°C, 5% CO_2_, with the media changed every two to three days.

### 2.3. Secretion of IL-1*β*, MCP-1, IP-10, and Cytotoxins by U118 MG Astrocytic Cells

U118 MG astrocytic cells were plated in 24-well plates at a concentration of 2 × 10^5^ cells/ml in one ml DMEM-F12 containing antibiotics and 5% CBS. One well was used for each experimental condition. For experiments performed in serum-free conditions, CBS was substituted with B-27 supplement with vitamin A (Fisher Scientific). The cells were incubated for 24 h to allow their adherence. CL at concentrations similar to levels detected in plasma from fasting humans (0.5-20 *μ*g/ml) [14] or its vehicle solution (1% ethanol) was added to culture media, followed by a 15 min incubation period. U118 MG astrocytic cells were treated with LPS (400 ng/ml), IL-1*β* plus IFN-*γ* (400 U/ml and 20 pg/ml, respectively), or their vehicle solutions (PBS). Neither of the vehicle solutions (PBS or 1% ethanol) had a significant effect on the viability of U118 MG astrocytic cells. In a subset of experiments, 30 min prior to the addition of CL, 10 *μ*M TAK-242 was added to the culture media of U118 MG astrocytes. Concentrations of MCP-1, IP-10, and IL-1*β* in supernatants were measured 48 h later using PeproTech ELISA development kits according to the manufacturer's instructions.

To study the effect of CL on secretion of cytotoxins by astrocytes, 400 *μ*l of collected U118 MG astrocytic cell supernatants were transferred to separate wells containing SH-SY5Y neuronal cells that had been plated 24 h earlier at a concentration of 4 × 10^5^ cells/ml in 400 *μ*l of DMEM-F12 containing antibiotics and 5% CBS. After 72 h incubation, neuronal cell viability was measured by the MTT assay. Viability of U118 MG astrocytic cells was also assessed by the MTT assay at the end of the 48 h incubation period with CL, stimuli, or TAK-242.

### 2.4. Assessing Cell Viability

Cell viability was monitored by the MTT assay, which measured the reduction of MTT to an insoluble purple formazan product by viable cells [26, 27]. Cells were incubated with MTT (0.5 mg/ml) at 37°C for one h in a 5% CO_2_ incubator. The resulting formazan crystals were dissolved by adding a volume of SDS (20% w/v)/N,N-dimethylformamide (50% v/v) solution equal to that of the culture medium present in the well, then incubating the plates for three h. Optical densities were measured at 570 nm using the FLUOstar Omega microplate reader.

### 2.5. Preparation of Synaptosomes

Crude synaptosomal fractions were obtained from brains of adult C57BL/6NCrl wild-type mice as previously described [28]. Briefly, a tissue grinder was used to homogenize a single brain in lysis buffer (pH 7.5) containing 0.32 M sucrose, 5 mM HEPES, and a protease inhibitor cocktail (Roche, Mississauga, ON, Canada). Homogenate was centrifuged 10 min at 1,000 g, at 4°C. The supernatant was collected and centrifuged 10 min at 12,000 g, at 4°C. The pellet containing crude synaptosomes was resuspended in PBS and stored at -80°C. The presence of the synaptic marker PSD-95 in the synaptosomal preparation was demonstrated using immunoblotting. Synaptosomes were stained with CellBrite® Fix 555 or Fix 640. Stained synaptosomal preparations were then dialyzed overnight in PBS at 4°C to remove the remaining dye from the preparation.

### 2.6. Phagocytic Activity of Human U118 MG Astrocytic Cells and Primary Murine Astrocytes

Phagocytic activity was assessed as previously described with minor modifications [29]. Human U118 MG astrocytic cells were plated in poly-D-lysine-coated, four-chambered Petri dishes at a density of 5 × 10^4^ cells/ml in 500 *μ*l DMEM-F12 containing antibiotics and 5% CBS. Murine astrocytes were plated at a density of 5 × 10^4^ cells/ml in DMEM containing antibiotics and 10% FBS. After 48 h, CL (20 *μ*g/ml) or its vehicle solution was added to cell cultures. Following 48 h incubation, culture media were replaced with 500 *μ*l of fresh media. 10 *μ*l of diluted synaptosomes suspension (1 : 5 stock) or one *μ*m diameter externally FITC-labeled latex beads (10 : 1 bead to cell ratio) were added, and cell cultures were incubated for two or one h, respectively, at 37°C. After two washes with one ml PBS, U118 MG astrocytic cell monocultures were visualized by adding 300 *μ*l of one *μ*g/ml Texas red membrane stain for five min followed by a single washing step with one ml PBS. Prior to imaging, bisbenzimide (two *μ*g/ml) nuclear stain was added to all wells in 500 *μ*l PBS. Since primary astrocyte cultures contain small number of microglia, GFAP immunostaining was performed to visualize astrocytes. Astrocyte cultures were fixed with 4% paraformaldehyde in PBS and permeabilized using 0.1% Triton X-100. Cells were washed twice with PBS, and a blocking solution containing 2.5% BSA and 0.02% Triton X-100 was added for one h at room temperature. Cells were incubated with an anti-GFAP antibody (1 : 2,000 dilution) overnight at 4°C in the same blocking solution. Astrocytes were washed three times with PBS and incubated with goat anti-rabbit CY5- or CY3-labeled antibodies (1 : 1,000 dilution) in a solution containing 2.5% BSA and 1% goat serum for one h at room temperature. Cells were washed with PBS and stained with bisbenzimide before imaging. Cells were imaged with a Zeiss AxioObserver.Z1 widefield epifluorescence microscope with Zen image acquisition software (version 2.0) to quantify fluorescence at an excitation/emission of 365/445 nm for bisbenzimide, 474/537 nm for the fluorescent beads, 620/670 nm for CY5, 545/605 nm for CY3, 585/615 nm for Texas red membrane stain, and 555/605 nm for CellBrite® Fix 555. The fluorescence intensity was calculated as a corrected total cell fluorescence (CTCF). The engulfment of fluorescent beads and synaptosomes was confirmed with a Leica TCS SPE-II confocal microscope (Leica Microsystems; Concord, ON, Canada). Images shown were generated by the accompanying LAS X software (version 3.5.5)

### 2.7. Immunoblotting GFAP in Lysates of U118 MG Cells

U118 MG astrocytic cells were plated and treated as previously described in Section 2.3. After 48 h, cell supernatants were collected, cells were washed once with PBS and lysed with radioimmunoprecipitation assay buffer (RIPA buffer: 150 mM sodium chloride, 50 mM Tris, 1% Triton X-100, 0.5% sodium deoxycholate, 0.1% sodium dodecyl sulfate (SDS), 1X cOmplete™ mini protease inhibitor cocktail, pH 8.0). The concentrations of proteins in cell lysates were measured using the Pierce™ bicinchoninic acid (BCA) protein assay (Fisher Scientific). Protein samples were diluted in RIPA buffer to equal concentrations (100 *μ*g/ml), diluted in an equal volume of Laemmli buffer (0.125 M Tris, 0.1 M DTT, 20% glycerol, 4% SDS, 0.004% bromophenol blue), and heated at 90°C for four min. Two *μ*g of protein was loaded into each well of the 12% polyacrylamide gel. Following electrophoresis, proteins were transferred onto nitrocellulose membranes. Immunostaining was performed after membranes were blocked with 5% skim milk powder in Tris-buffered saline- (TBS-) Tween (150 mM NaCl, 10 mM Tris, 0.2% Tween-20, pH 8.0). Overnight incubation at 4°C with rabbit anti-GFAP antibodies (1 : 1,000 dilution) and rabbit anti-*β*-actin antibodies (1 : 1,000 dilution) diluted in 5% w/v nonfat powdered milk in TBS-Tween was followed by exposure to the anti-rabbit HRP-linked antibodies (1 : 1,000 dilution) for one h, and the SuperSignal™ West Pico PLUS enhanced chemiluminescence (ECL) reagent for five min. Immunostained proteins were visualized using the Fluorchem® FC2 image system with AlphaView Q 3.2.2.0 gel acquisition software (Cell Biosciences, Santa Clara, CA, USA). The gel analysis function of ImageJ 1.53a software (National Institute of Health, USA) was used to quantify data.

### 2.8. Expression of TLR 4 by U118 MG Astrocytic Cells

Expression of TLR 4 by U118 MG astrocytic cells was measured as previously described [27]. Cells were plated in 96-well plates at 2 × 10^5^ cells/ml in 250 *μ*l DMEM-F12 containing antibiotics and 5% CBS, followed by a 24 h incubation period to allow their adherence. Two wells were used for each experimental condition. CL (20 *μ*g/ml) or its vehicle solution was added to culture media, followed by LPS 15 min later. After a 48 h incubation period, supernatants were removed, and cells were fixed by air drying for 30 min. The plates were incubated at room temperature with 3% BSA in PBS for two h, washed three times with PBS, and incubated with monoclonal anti-human TLR 4 antibodies (1 : 100 dilution) in 3% BSA in PBS for one h. Plates were washed thrice with PBS and incubated with goat anti-mouse IgG alkaline phosphatase-conjugated antibodies (1 : 3,000 dilution) in 3% BSA in PBS for one h. After washing three more times with PBS, one mg/ml of phosphatase substrate in 0.1 M diethanolamine buffer (pH 9.8) was added to cell cultures. Optical densities at 405 nm were measured two h later using a FLUOstar Omega microplate reader. Optical density signal in each well was normalized to protein concentration in the same well as measured by the BCA assay.

### 2.9. Data Analysis

Data were analyzed using either (1) Student's *t*−test or (2) randomized block design one-way analysis of variance (ANOVA), followed by Dunnett's or Tukey's post-hoc test. Herein, independent experiments are defined as assays performed on different days. Data are presented as means ± standard error of the mean (SEM). Statistical significance was established at *P* < 0.05.

## 3. Results

### 3.1. CL Reduces Human U118 MG Astrocyte-Like Cell-Mediated Cytotoxicity towards SH-SY5Y Neuronal Cells

**Figure 1:**
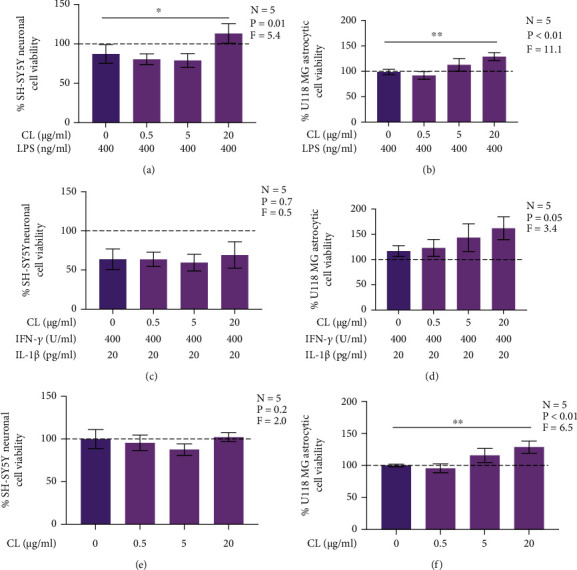
The effect of CL on cytotoxicity of human U118 MG astrocytic cells towards human SH-SY5Y neuronal cells. Increasing concentrations of CL alone or its vehicle solution was added to U118 MG astrocytic cell cultures (e, f), or CL was added 15 min before exposure of astrocytic cells to LPS (400 ng/ml) (a, b) or a combination of IFN-*γ* (400 U/ml) plus IL-1*β* (20 pg/ml) (c, d). After 48 h, U118 MG cell supernatants were transferred onto SH-SY5Y neuronal cell cultures, and the viability of astrocytic cells (b, d, f) was measured by the MTT assay. Following 72 h exposure to U118 MG astrocytic cell supernatants, SH-SY5Y viability (a, c, e) was measured using the MTT assay. All data (presented as means ± SEM) in this figure are normalized against cell viability values (shown as dashed lines) obtained from U118 MG cells exposed to the CL solvent (1% ethanol) only (b, d, f) or SH-SY5Y cells incubated in supernatants from U118 MG astrocytic cells that have been exposed to the CL solvent only (a, c, e). ^∗^*P* < 0.05 and ^∗∗^*P* < 0.01 according to Dunnett's post-hoc test. *P* and *F* values for the one-way randomized blocks ANOVA are shown.

Inflammatory mediators released by reactive astrocytes can have toxic effects on surrounding neurons contributing to neurodegeneration observed in AD brains [30]. A previous study demonstrated CL reduced the cytotoxicity of microglia-like cells towards neuronal cells [15]; therefore, we investigated whether this phospholipid could reduce the release of cytotoxins by astrocytic cells.  Figure 1 illustrates reduced viability of SH-SY5Y cells incubated in supernatants from U118 MG astrocytic cells that have been stimulated with LPS (Figure 1(a)) or IFN-*γ* plus IL-1*β* (Figure 1(c)) compared to neuronal cells exposed to supernatants from unstimulated astrocytic cells (Figure 1(e)). CL added at 20 *μ*g/ml prior to stimulation of U118 MG astrocytic cells with LPS reversed the cytotoxic effects of their supernatants (Figure 1(a)). This inhibitory effect of CL was not due to reduced viability of U118 MG astrocytic cells (Figure 1(b)). CL also did not increase viability of SH-SY5Y cells exposed to supernatants from unstimulated U118 MG cells (Figure 1(e)). CL did not inhibit cytotoxicity of IFN-*γ* plus IL-1*β*-stimulated U118 MG astrocytic cells towards SH-SY5Y neuronal cells (Figure 1(c)) and did not affect viability of IFN-*γ* plus IL-1*β*-stimulated U118 MG astrocytic cells (Figure 1(d)). CL had small but significant positive effects on the viability of LPS-stimulated and unstimulated U118 MG cells (Figures 1(b) and 1(f)).

### 3.2. CL Upregulates the Phagocytic Activity of Human U118 MG Astrocytic Cells and Primary Murine Astrocytes

**Figure 2:**
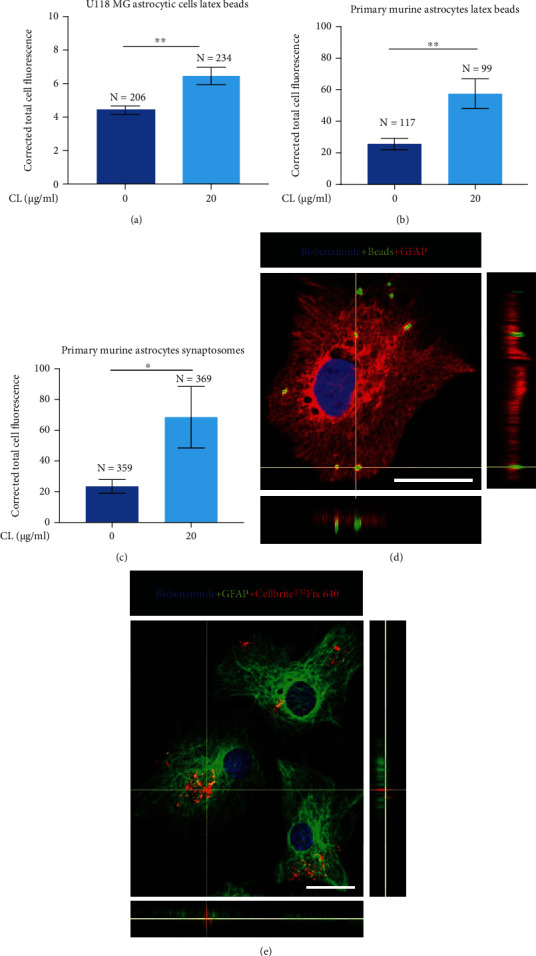
The effect of CL on the phagocytic activity of U118 MG astrocytic cells (a) and primary murine astrocytes (b, c). CL (20 *μ*g/ml) or its vehicle solution was added to U118 MG cells or primary murine astrocyte cultures for 48 h, followed by incubation with fluorescent latex beads (a, b) or synaptosomes (c). The fluorescence intensities of 99-369 randomly selected cells (means ± SEM) from four independent experiments performed on different days are presented as corrected total cell fluorescence. The selection of cells and analysis of fluorescence were performed in a blinded manner. ^∗^*P* < 0.05 and ^∗∗^*P* < 0.01 according to the unpaired Student's *t*-test. Representative confocal images confirming the uptake of FITC-labeled latex beads (d) and CellBrite™ Fix 640-labeled synaptosomes (e) by GFAP-positive primary murine astrocytes are shown; the scale bar represents 25 *μ*m.

Since CL is known to upregulate the phagocytic activity of human and murine microglia [15, 17], we evaluated if this phospholipid could have a similar effect on astrocytes. Compared to cells treated with the vehicle solution only, CL alone upregulated phagocytosis of fluorescent latex beads by U118 MG astrocytic cells (Figure 2(a)) and primary murine astrocytes (Figure 2(b)). In addition, phagocytosis of adult murine brain-derived synaptosomes by murine astrocytes was also upregulated by CL (Figure 2(c)). Engulfment of beads and synaptosomes by GFAP-positive primary astrocytes was confirmed by confocal microscopy (Figures 2(d) and 2(e)).

### 3.3. CL Downregulates the LPS-Induced Increase in GFAP Expression and, in Combination with LPS, Upregulates the Secretion of IL-1*β* by U118 MG Astrocytic Cells

**Figure 3:**
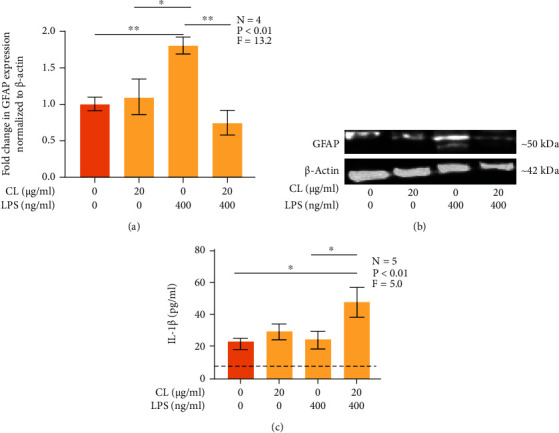
The effect of CL on the expression of GFAP (a) and secretion of IL-1*β* (b) by human U118 MG astrocytic cells. CL (20 *μ*g/ml) or its vehicle solution was added to U118 MG cell cultures 15 min before exposure to LPS (400 ng/ml) or the vehicle solution. After 48 h, immunoblotting was performed using cell lysates (a), and IL-1*β* concentration in cell supernatants was measured by an ELISA (b). Data (means ± SEM) are expressed as fold change in protein levels compared to control cells exposed to growth medium only and are normalized to *β*-actin (a). The detection limit of the ELISA is presented as the dotted line (b). ^∗^*P* < 0.05 and ^∗∗^*P* < 0.01 according to Tukey's post-hoc test. *P* and *F* values for the one-way randomized blocks ANOVA are shown. Representative bands of immunostained GFAP and *β*-actin from each treatment group are shown (c). Uncropped immunoblot images are provided as Supplementary Figure 1.

In chronic neuroinflammation, astrocytes become reactive. This phenotypic and functional state of astrocytes is characterized by an upregulated expression of the intermediate filament protein GFAP [31]. We investigated whether CL prevents upregulation of GFAP protein levels in U118 MG astrocytic cells following their exposure to LPS or IFN-*γ* plus IL-1*β*, which were used as potent immune stimuli acting through differing receptors linked to distinct signaling pathways. Figures 3(a) and 3(c) demonstrate that LPS alone upregulated the expression of GFAP by U118 MG astrocytic cells compared to unstimulated cells and cells treated with CL alone. The addition of CL significantly inhibited this LPS-induced increase in the GFAP expression (Figure 3(a)).

The observation that CL inhibited LPS-induced upregulation of GFAP implicated TLR 4 as a possible astrocytic target of extracellular CL. Activation of TLR 4 has been shown to upregulate the secretion of IL-1*β* by human astrocytes [22]. We hypothesized that CL acting on TLR 4 would affect the secretion of IL-1*β* by U118 MG astrocytic cell.  Figure 3(b) demonstrates that while CL alone did not induce the secretion of IL-1*β* by U118 MG astrocytic cells, its combination with LPS caused significant upregulation of IL-1*β* secretion compared to unstimulated cells as well as cells stimulated with LPS alone.

### 3.4. CL Downregulates the TLR 4 Expression by U118 MG Astrocytic Cells

**Figure 4:**
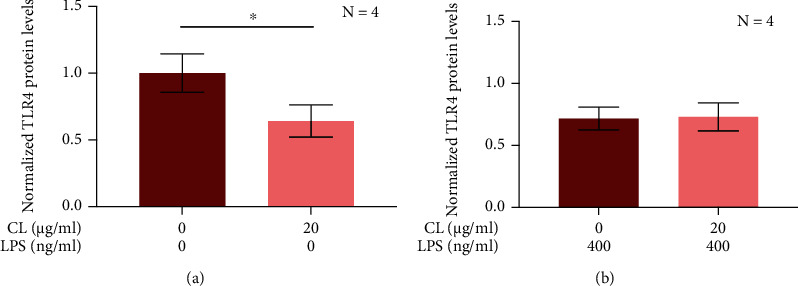
The effect of CL on the expression of TLR 4 by unstimulated (a) and LPS-stimulated (b) human U118 MG astrocytic cells. CL (20 *μ*g/ml) or its vehicle solution was added to U118 MG astrocytic cell cultures 15 min before exposure to LPS (400 ng/ml) or its vehicle solution. After 48 h, TLR 4 protein levels were measured by immunostaining. The resulting chemiluminescence signal was normalized to protein concentration in each well. Data (means ± SEM) are presented as a ratio of normalized chemiluminescence signal to that obtained from untreated control cells. ^∗^*P* < 0.05 according to the paired Student's *t*-test.

The Myd88-independent signaling associated with TLR 4 is known to induce endocytosis, which in turn leads to degradation of this receptor [32, 33]; therefore, we measured the expression of TLR 4 by U118 MG astrocytic cells following their exposure to CL.  Figure 4(a) demonstrates that the TLR 4 levels were significantly lower in U118 MG cells exposed to extracellular CL compared to cells treated with the vehicle solution only. However, pretreatment with extracellular CL did not further downregulate the expression of TLR 4 by LPS-stimulated U118 MG astrocytic cells (Figure 4(b)).

### 3.5. CL Induces the Secretion of MCP-1 by Human U118 MG Astrocytic Cells in Serum-Containing Medium

**Figure 5:**
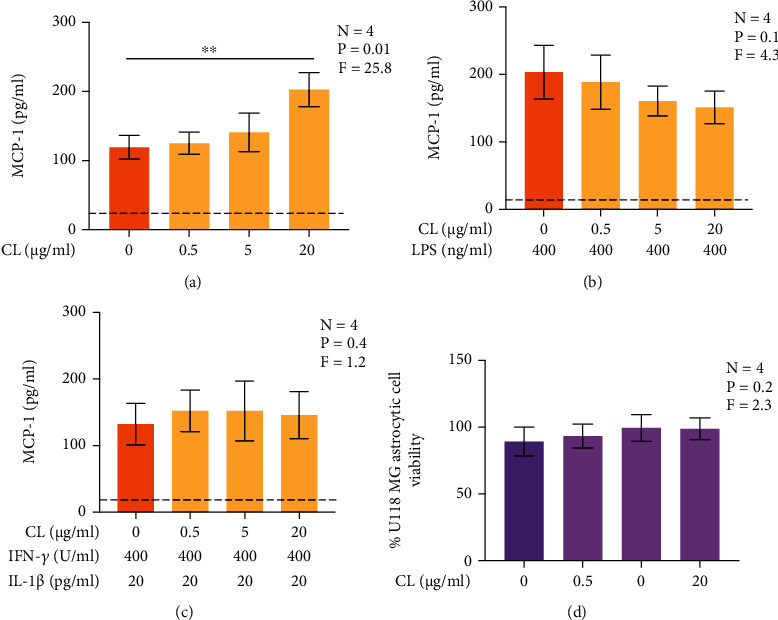
The effect of CL on human U118 MG astrocytic cell MCP-1 secretion (a)–(c) and their viability (d) in serum-containing medium. Increasing concentrations of CL or its vehicle solution was added to U118 MG astrocytic cell cultures (a, d), or CL was added 15 min before exposure to LPS (400 ng/ml) (b) or a combination of IFN-*γ* (400 U/ml) plus IL-1*β* (20 pg/ml) (c). After 48 h, concentrations of MCP-1 in cell supernatants were measured by an ELISA, and the viability of U118 MG astrocytic cells was measured by the MTT assay. Data are presented as means ± SEM. Cell viability in (d) is normalized to values obtained from U118 MG cultures not exposed to CL or its vehicle solution. ^∗∗^*P* < 0.01 according to Dunnett's post-hoc test. *P* and *F* values for the one-way randomized blocks ANOVA are shown. The detection limit of the ELISA is presented as the dotted line.

MCP-1 and IP-10 are known to be secreted, along with other proinflammatory cytokines such as IL-1*β*, IL-6, and TNF-*α*, following the activation of astrocyte TLR 4 [20–22]. They are also upregulated in AD brain tissues and cerebrospinal fluid, respectively [34, 35]. We hypothesized that CL upregulates the secretion of MCP-1 and IP-10 by human astrocytic cells since similar effect has already been reported for human microglia-like cells, which also express TLR 4 [17].  Figure 5(a) shows that CL alone upregulated the secretion of MCP-1 by U118 MG astrocytic cells. Furthermore, although not significant, there was a trend towards a decrease in MCP-1 secretion when CL was added to LPS-stimulated U118 MG astrocytic cells (Figure 5(b)). CL had no effect on the IFN-*γ* plus IL-1*β*-induced secretion of MCP-1 or the viability of U118 MG astrocytic cells under all conditions of this experiment (Figures 5(c) and 5(d)). CL alone had no effect on the secretion of IP-10 by U118 MG astrocytic cells (data not shown). Preliminary data obtained by a multiplex assay indicated that exposure of U118 MG cells to CL alone for 48 h could also induce secretion of low levels of IL-1*β*, IL-6, and TNF-*α*; however, these effects were not significant with the limited numbers of observations made (data not shown); therefore, subsequent experiments focused on secretion of MCP-1 by U118 MG cells.

### 3.6. CL Regulates MCP-1 Secretion in Serum-Free Conditions

**Figure 6:**
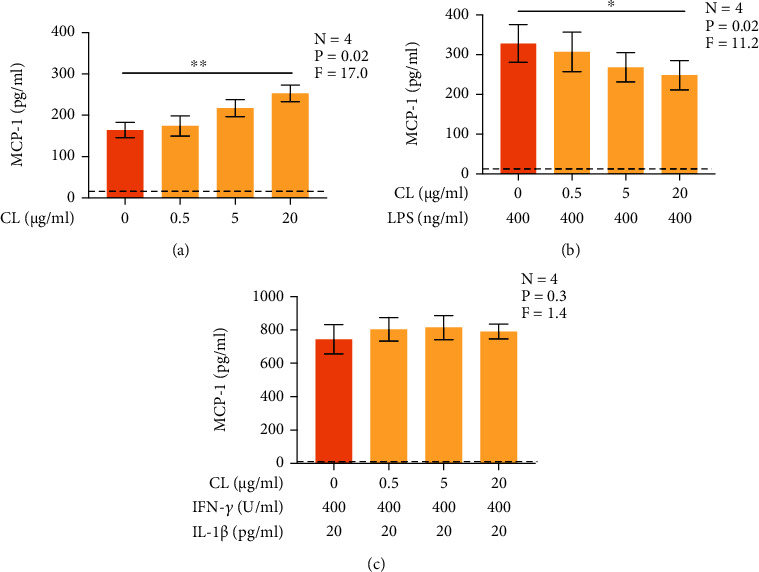
The effect of CL on human U118 MG astrocytic cell MCP-1 secretion in serum-free medium. Increasing concentrations of CL or its vehicle solution was added to U118 MG astrocytic cell cultures (a), or CL was added 15 min before exposure to LPS (400 ng/ml) (b), a combination of IFN-*γ* (400 U/ml) plus IL-1*β* (20 pg/ml) (c). After 48 h, the concentrations of MCP-1 in cell supernatants were measured by an ELISA. Data are presented as means ± SEM. ^∗^*P* < 0.05 and ^∗∗^*P* < 0.01 according to Dunnett's post-hoc test. *P* and *F* values for the one-way randomized blocks ANOVA are shown. The detection limit of the ELISA is presented as the dotted line.

To investigate whether the carrier protein of CL, which is present in serum, including CBS [14], was required for CL to regulate immune functions of astrocytes, we assessed the effects of CL on MCP-1 secretion by U118 MG astrocytic cells in serum-free conditions. CL alone upregulated the secretion of MCP-1 by U118 MG astrocytic cells (Figure 6(a)). In addition, CL significantly reduced the secretion of MCP-1 by LPS-stimulated U118 MG astrocytic cells (Figure 6(b)) but had no effect on the secretion of MCP-1 by IFN-*γ* plus IL-1*β*-stimulated U118 MG astrocytic cells (Figure 6(c)). Under neither of these three experimental conditions did CL reduce the viability of U118 MG astrocytic cells (data not shown).

### 3.7. CL Induces the Secretion of MCP-1 by Human U118 MG Astrocytic Cells in a TLR 4-Dependent Manner

**Figure 7:**
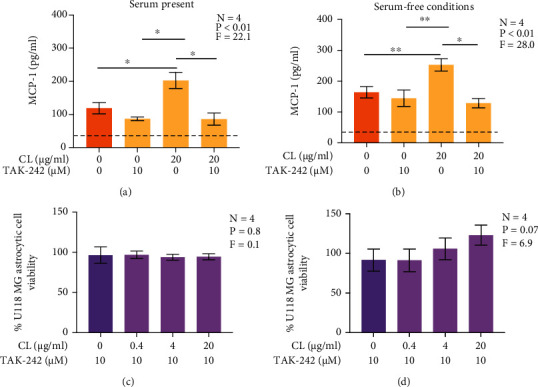
The effect of TLR 4-specific antagonist TAK-242 on CL-induced secretion of MCP-1 by human U118 MG astrocytic cells and their viability in serum-containing (a, c) and serum-free (b, d) media. TAK-242 (10 *μ*M) was added to U118 MG astrocytic cells 30 min before their exposure to CL. After 48 h, the concentration of MCP-1 in cell supernatants was measured by an ELISA, and the viability of U118 MG astrocytic cells was measured by the MTT assay. Data are presented as means ± SEM. Cell viability in (c, d) is normalized to values obtained from U118 MG cultures not exposed to CL, TAK-242, or their vehicle solutions. ^∗^*P* < 0.05 and ^∗∗^*P* < 0.01 according to Tukey's post-hoc test. *P* and *F* values for the one-way randomized blocks ANOVA are shown. The detection limit of the ELISA is presented as the dotted line.

In silico and in vitro studies with nonastrocytic cells have implicated TLR 4 as one of the receptors binding extracellular CL [16, 17, 24]. Our data above showing the selective inhibitory effects of CL on LPS-stimulated cells and downregulation of TLR 4 expressions by U118 MG cells exposed to CL indicated the interaction between this phospholipid and astrocytic TLR 4. To confirm this directly, we investigated whether the CL-induced secretion of MCP-1 secretion by U118 MG astrocytic cells was inhibited by a TLR 4-specific antagonist TAK-242. Similar to the experiments described above, CL alone upregulated the secretion of MCP-1 by U118 MG astrocytic cells in both serum-containing (Figure 7(a)) and serum-free (Figure 7(b)) medium. These effects of CL were inhibited by TAK-242 (Figures 7(a) and 7(b)). TAK-242 on its own had no significant effect on MCP-1 secretion by U118 MG astrocytic cells. TAK-242 alone or in combination with CL did not affect the viability of U118 MG astrocytic cells according to the MTT assay in both serum-containing (Figure 7(c)) and serum-free (Figure 7(d)) medium.

## 4. Discussion

In addition to its established intracellular roles as a mitochondrial membrane phospholipid, CL acts as a bioactive molecule in the intercellular space, where it has been shown to modulate the functions of immune cells, including macrophages in the periphery and microglia in the CNS [15–17, 24]. Our study is the first to demonstrate that extracellular CL modulates select immune functions of astrocytes, a CNS cell type also known to participate in regulation of neuroimmune responses. We demonstrate that CL upregulates phagocytic activity of primary murine astrocytes and has complex modulatory effects on human astrocytic cell activation status assessed by the GFAP expression and phagocytic activity as well as their secretion of cytokines and cytotoxins. With regard to the complex immunoregulatory effects of CL on astrocytes, this phospholipid (1) downregulates secretion of cytotoxins and MCP-1 by LPS-stimulated human U118 MG astrocytic cells, (2) upregulates phagocytic activity of human astrocytic cells and primary murine astrocytes, (3) prevents upregulation of GFAP by LPS-stimulated cells, and (4) induces secretion of the cytokines MCP-1 and IL-1*β*, but not IP-10. Our study also implicates astrocyte TLR 4 as one of the molecular targets for extracellular CL.

Similar to microglia, astrocytes secrete cytokines and cytotoxins in response to immune stimuli, as well as display phagocytic activity [36, 37]. Our observation that CL inhibits the secretion of cytotoxins by LPS-activated U118 MG astrocytic cells is similar to previous studies showing that CL reduces the secretion of cytotoxins by human THP-1 monocytic cells used as models of microglia [15, 17]. The upregulated secretion of cytotoxins by U118 MG astrocytic cells can be attributed to their transition from a homeostatic to a reactive state (for a review, see [3]). Our data showing the upregulated GFAP expression by U118 MG astrocytic cells in response to LPS stimulation supports this hypothesis. Since extracellular CL inhibits the secretion of cytotoxins and MCP-1, and blocks upregulations of GFAP when added before LPS, this phospholipid may prevent the transition of astrocytes from a homeostatic to a reactive state, which can have beneficial effect under neuroinflammatory conditions in the CNS.

Phagocytic activity of glial cells is crucial for resolution of neuroinflammation, and dysregulated phagocytosis facilitates neurodegeneration [5, 9, 38–40]. Microglia are established phagocytes of the CNS, and regulation of their phagocytic activity is well documented (for a review, see [41]); meanwhile, very little is known about molecules that modulate phagocytic activity of astrocytes, even though their ability to engulf particles is becoming widely recognized [42, 43]. Our observation that CL upregulates the phagocytic activity of U118 MG astrocytic cells and primary murine astrocytes indicates yet another beneficial function of this phospholipid. This phagocytosis-inducing effect of CL on astrocytes is similar to the previously reported upregulation of phagocytosis by CL and liposomes containing CL in microglia and RAW264.7 murine macrophages, respectively [15–17]. While in these previous studies, the phagocytic activity was assessed by measuring engulfment of latex beads or CL-containing liposomes only, and we demonstrate CL also that upregulates the engulfment of murine brain synaptosomes by murine primary astrocytes, indicating this observation is physiologically relevant and not an artifact related to phagocytosis of latex beads.

As mentioned above, extracellular CL has already been shown to modulate the secretion of cytokines by microglia and peripheral macrophages. We extend the list of cell types that CL affects by demonstrating this phospholipid induces the secretion of MCP-1 and IL-1*β* by U118 MG astrocytic cells. Our observation that a combination of CL and LPS is required for significant upregulation of IL-1*β* secretion by human astrocytic cells is in line with a study by Dozio and Sanchez [22] who observed that LPS on its own caused only moderate increase in secretion of this cytokine by human primary astrocytes. While CL alone has been shown to upregulate the release of IP-10 by human THP-1 monocytic cells [17], the secretion of this cytokine is not induced in U118 MG astrocytic cells. Thus, the effects of CL on inflammatory cytokine secretion appear to be cell-type specific [44, 45]. Furthermore, CL exhibits its immune-modulating effects in a cell activation state-dependent manner. For example, our study demonstrates that CL reduces the secretion of MCP-1 by immune-stimulated astrocytic cells. Yet, when administered to unstimulated cells, CL induces the secretion of this cytokine. Similarly, differential effects of CL on immune stimulated and unstimulated macrophages and microglia have been reported previously for MCP-1 and IP-10 [15, 24].

Most of the experiments in this study used cell culture medium that included CBS. Serum is known to contain the natural carrier protein of CL, *β*2-glycoprotein 1 (also known as apolipoprotein H), which may be required for certain physiological functions of extracellular CL [14]. Therefore, we investigated whether the effects of CL observed in this study were dependent on the presence of serum in the media, as we would not expect this plasma protein to be present in the extracellular space of the brain parenchyma. We demonstrate that the effects of CL on the secretion of MCP-1 by U118 MG astrocytic cells do not depend on the presence of CBS, indicating that immunomodulatory effects of CL do not require the presence of this specific carrier protein. Our observations are similar to a previous study demonstrating that the TLR 4 agonist LPS does not require carrier proteins present in CBS to induce the secretion of cytokines from IC-21 murine macrophages [46]. However, to the best of our knowledge, the necessity of a carrier protein has not been assessed before for any of the extracellular effects of CL.

The role of TLR 4 as one of the receptors mediating the effects of extracellular CL on astrocytes is supported by the following of our observations: (1) CL inhibits secretion of cytotoxins and MCP-1 by astrocytes stimulated with the TLR 4 ligand LPS, but not a combination of IFN-*γ* plus IL-1*β*, which act on different receptors, (2) CL in combination with LPS induces secretion of IL-1*β*, which is linked to TLR 4 activation [22], (3) CL on its own downregulates the astrocyte TLR 4 expression, and (4) CL-induced secretion of MCP-1 is blocked by the TLR 4-specific antagonist TAK-242. These observations support previous studies in which TLR 4 has already been demonstrated to mediate effects of extracellular CL on microglia, macrophages, and monocytic cells. The knockout of TLR 4 in bone marrow-derived murine macrophages abolishes the CL-induced increase in TNF-*α* secretion [24]. Furthermore, Wenzel et al. [17] demonstrate that TLR 4 mediates CL-induced MCP-1 secretion by primary murine microglia and THP-1 monocytic cells, as well as their phagocytic activity. Similar to our observations that CL reduces the expression of TLR 4, Marinelli et al. [47] demonstrate that TLR agonists, including the potent TLR 4 agonist LPS, modulate the expression of TLR 4 on microglia and to a lesser extent astrocytes. Taken together, our data may indicate that CL inhibits the effects of LPS by reducing the number of TLR 4 molecules expressed by astrocytes, which is a phenomenon exhibited by other TLR 4 agonists such as 1Z105 and 1Z88 [48]. Even though there is strong evidence to suggest that TLR 4 is the molecular target of CL on astrocytes as well as other cell types, it is possible that the inhibitory effects of CL on the LPS-induced release of MCP-1 and cytotoxins by astrocytic cells are mediated by other mechanisms. For example, CL could modulate the production of microRNAs that inhibit signaling events downstream of TLR 4 activation, which in turn reduces cytokine secretion (for a review, see [49]).

Overall, or study indicates that extracellular CL could have beneficial immunomodulatory effects on astrocytes by inhibiting their transition to a reactive state, reducing secretion of cytotoxins and select inflammatory mediators, and upregulating their phagocytic activity. These effects are observed by using cultured astrocytic cells and, therefore, will require in vivo confirmation before CL can be considered as a biotherapeutic agent for the treatment of neuroinflammatory disorders such as AD. This is especially important since astrocytes are known to exhibit different phenotypes and functional responses in the presence of microglia [50].

## 5. Conclusions

Previous studies have demonstrated that CL regulates the cytokine secretion and phagocytic activity of immune cells, including peripheral macrophages and microglia [15–17]. Our study extends the list of cell types that CL interacts with by revealing this phospholipid modulates select immune functions of astrocytes, and that this activity is mediated, at least partially, by TLR 4. Importantly, our data demonstrate that extracellular CL acts even in the absence of its natural carrier protein, *β*2-glycoprotein 1, indicating that CL could be bioactive in the brain where this protein is not present under physiological conditions. CL and CL-containing liposomes have already been suggested as possible therapeutic agents for treatment of inflammatory disorders in peripheral tissues as well as CNS [15–17]. Our study supports the potential of this therapeutic strategy by identifying astrocytes as one the key cell types beneficially affected by extracellular CL.

## Data Availability

The data that support the findings of this study are available from the corresponding author upon request.

## Supplementary Materials

Supplementary Materials Supplementary Figure 1: representative immunoblots showing the effects of CL on the expression of GFAP and *β*-actin by human U118 MG astrocytic cells.
