# Supplementary material for: Extracellular Cardiolipin Modulates Select Immune Functions of Astrocytes in Toll-Like Receptor (TLR) 4-Dependent Manner
Source: Mediators Inflamm. 2022 Mar 25;2022:9946439. doi: 10.1155/2022/9946439 (PMC8975658; doi:10.1155/2022/9946439)
Supplement: Supplementary Materials — Supplementary Figure 1: representative immunoblots showing the effects of CL on the expression of GFAP and β-actin by human U118 MG astrocytic cells. [file 9946439.f1.docx]

**Supplementary Figure 1**

Representative immunoblots showing the effects of CL on the expression of GFAP and β-actin by human U118 MG astrocytic cells (see **Fig. 3C** of the main manuscript for quantitative data).

1, vehicle solutions; 2, CL (20 µg/ml); 3 - LPS (400 ng/ml); 4 LPS + CL; X, protein bands from samples that are not relevant to this study.
